# Financial Fraud and Deception in Aging

**DOI:** 10.20900/agmr20230007

**Published:** 2023-09-11

**Authors:** Natalie C. Ebner, Didem Pehlivanoglu, Alayna Shoenfelt

**Affiliations:** 1 Department of Psychology, University of Florida, Gainesville, FL 32611, USA; 2 Florida Institute for Cybersecurity Research, University of Florida, Gainesville, FL 32611, USA; 3 Florida Institute for National Security, University of Florida, Gainesville, FL 32611, USA; 4 Institute on Aging, University of Florida, Gainesville, FL 32611, USA; 5 Center for Cognitive Aging and Memory, University of Florida, Gainesville, FL 32610, USA

**Keywords:** deception detection, financial exploitation, fraud, elder maltreatment, aging, decision making, cognitive decline, socioemotional function, brain structure/function, Alzheimer’s Disease

## Abstract

Financial exploitation among older adults is a significant concern with often devastating consequences for individuals and society. Deception plays a critical role in financial exploitation, and detecting deception is challenging, especially for older adults. Susceptibility to deception in older adults is heightened by age-related changes in cognition, such as declines in processing speed and working memory, as well as socioemotional factors, including positive affect and social isolation. Additionally, neurobiological changes with age, such as reduced cortical volume and altered functional connectivity, are associated with declining deception detection and increased risk for financial exploitation among older adults. Furthermore, characteristics of deceptive messages, such as personal relevance and framing, as well as visual cues such as faces, can influence deception detection. Understanding the multifaceted factors that contribute to deception risk in aging is crucial for developing interventions and strategies to protect older adults from financial exploitation. Tailored approaches, including age-specific warnings and harmonizing artificial intelligence as well as human-centered approaches, can help mitigate the risks and protect older adults from fraud.

## EXPLOITATION RISK AND DECEPTION IN AGING

Financial exploitation among older adults is a significant concern that severely impacts the fastest-growing segment of the population in industrialized nations, harming victims and society at large. Financial exploitation refers to the misuse or illegal acquisition of an older person’s funds, assets, or property, typically by someone in a position of trust and with malicious intent. It represents one of the most common forms of elder maltreatment [1,2]. Consequences from financial fraud can be devastating, both financially and emotionally, especially among older adults, including increased dependence, impoverished living conditions, decline in well-being, greater rates of hospitalization and long-term care admissions, poor physical and mental health, and even morbidity and mortality [3–5]. While people from any age group can be scammed, older adults experience greater monetary losses from fraud relative to younger adults [6]. According to the Federal Bureau of Investigation (FBI) [7], in 2021 for example there were 92,371 older victims of fraud resulting in $1.7 billion in losses, which was a 74% increase in losses compared to 2020. These high numbers of elderly victims and the dire consequences from financial abuse on their health and well-being highlight the significance of the problem, while not even accounting for significant underreporting [8,9], due to feelings of embarrassment, fear of losing independence, or lack of awareness by the victim [10].

Perpetrators of financial exploitation often are family members, caregivers, friends, and neighbors, but perpetrators can also be strangers. Perpetrators use a variety of psychological influences (i.e., weapons of influence [11]) and exploitation tactics, including coercion, manipulation, undue influence, and deception to gain control over an older adult’s assets [2,12]. To date, a variety of risk factors for vulnerability to fraud have been identified including cognitive decline and impaired decision making, social isolation, and dependence on others for activities of daily living as well as lack of experience and familiarity with financial matters among older adults [13]. Focused research is underway to comprehensively delineate cognitive, socioemotional, and neurobiological changes with age that contribute to particular risk profiles for uncovering and surveilling susceptibility to deception and exploitation risk in aging [14].

As noted, deception is a frequent and effective technique used in exploitation. The majority of current research studies on deception focuses on lie detection during interpersonal communication (e.g., how well people distinguish between truth and lie tellers; for meta-analytical reviews, see [15,16]). Deceptive contexts, however, are more diverse and complex than currently reflected in most research and extend across life domains (e.g., health, finances, relationships) and interaction fora (e.g., can occur face-to-face, over the phone, and increasingly over the internet in our globally connected, digital world) as well as include email/voice/text phishing, identity theft, fake news and hoaxes, false ads, deep fakes, consumer/health fraud, cryptocurrency/investment scam, and romance or gambling scams [7,17].

## INTERPLAY OF PERSON AND CONTEXT VARIABLES

**Figure 1.**
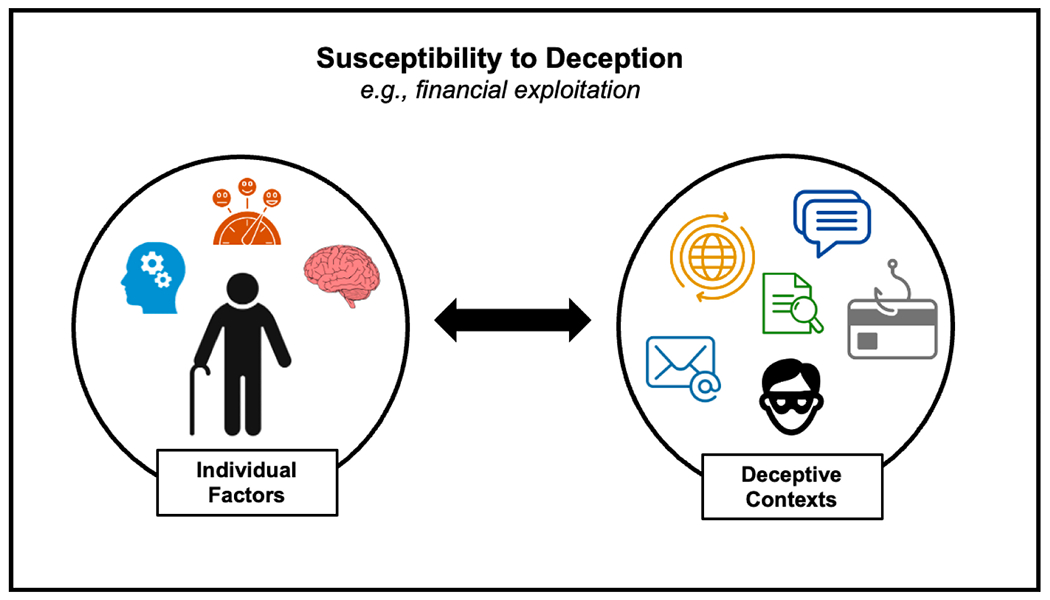
Schematic conceptual framework that considers interindividual differences in cognitive, socioemotional, and neurobiological influences in their interplay with characteristics of deceptive contexts on susceptibility to deception and exploitation risk.

Emerging evidence supports that, while deception detection is barely above chance [15], both characteristics of the individual confronted with a deception and the nature of the deceptive messages/information (e.g., content, semantic, ambiguity) influence whether deception is detected [14,18]. In fact, multiple individual factors have been identified that contribute to deception risk (see Figure 1 for a graphical overview; see also our recently proposed Biopsychosocial Model of Deception Risk in Aging [14]). In particular, this multifaceted conceptual framework considers cognitive, socioemotional, and neurobiological influences, and their interplay, on the aging decision maker in the context of deception detection and exploitation risk.

## INDIVIDUAL FACTORS

Regarding ***cognitive*** influences, processing speed, working memory, and executive functions decline with age [19,20] with demonstrated impact on how and how much information is processed and how and what decisions are made. More specifically, intuitive decision making remains relatively preserved in late life [21], while systematic information processing becomes less efficient with age [22]. This greater reliance on heuristics vs. deliberation has been shown to increase susceptibility to deception in aging [23]. Indeed, declines in analytical reasoning were associated with reduced detection of false news stories with greater age among older adults [24]. Additionally, low memory function was associated with greater susceptibility to email-based phishing in older adults with this effect demonstrated both in the laboratory as well as in real-life deception paradigms [25], and particularly pronounced among older adults at heightened risk for developing Alzheimer’s Disease (i.e., carriers of the apolipoprotein ε4 (APOE4) risk allele) [26]. This finding contributes to growing evidence that Alzheimer’s Disease and related dementias (ADRD) are associated with impaired (financial) decision making and heightened deception risk [27]; financial exploitability may even serve as an early detection measure of cognitive decline and ADRD [28,29].

Given the transactional nature of deception, ***socioemotional*** influences on deception risk in aging also play a crucial role. In particular, some research suggests that negative affect enhances deception detection (e.g., better lie detection [30]), while positive affect impairs it (e.g., decreased skepticism [31]). Higher positive affect was also associated with reduced ability to detect fake news with greater chronological age among older adults [24] (see [25], for the effect of positive affect on email phishing susceptibility in aging). Similarly, higher emotional intelligence (e.g., enhanced perspective taking abilities) promoted deception detection [32,33] and reduced susceptibility to scams [34]. Additionally, lack of social support and loneliness exacerbate deception risk, particularly among older adults [35]. Social isolation during the COVID-19 pandemic has led to increased reliance on online platforms [36] and there is evidence that particularly older adults with lower digital literacy are more vulnerable to deceptive messages [37,38], including fraudulent emails and robocalls impersonating health organizations [39,40], as well as misinformation via fake news [24].

Furthermore, ***neurobiological*** mechanisms, including psychophysiology, peripheral and brain chemistry, and brain structure/function play a critical role in deception detection in aging. For example, greater interoceptive awareness, reflecting the ability to accurately read internal physiological signals (e.g., “gut feelings” [41,42]), was correlated with greater physiological arousal to liars than truth-tellers and improved subsequent deception detection [43] and more rejection of unfair offers in financial decision making [44,45]. With particular relevance to aging, recent evidence supports that with greater chronological age among older adults greater interoceptive awareness was associated with better deception detection [46]. This effect was present both in lie detection and in phishing email detection [46,47]. These findings support interoceptive awareness as a relevant factor for interventions aimed at enhancing deception detection abilities among older adults.

Also, structural and functional changes in the brain with age have been associated with declining deception detection and greater risk for financial exploitation. In particular, older adults who had become victims of financial fraud in real life compared to older adults who had successfully avoided exploitation had both lower cortical volume in insula, a brain region that integrates internal signals with environmental cues [48], and lower volume in superior temporal cortex, a brain region in the default mode network important for social reasoning [49]. These exploited individuals also showed reduced functional connectivity within the default mode network, while increased functional connectivity between the default mode and the salience networks [50]; both networks implicated in memory functioning [49], financial decision making [51], and impression formation [52], crucial processes for deception detection [14,53]. Recent work has also demonstrated that self-reported financial exploitation in old age is associated with whole-brain functional connectivity differences involving the hippocampus, insula, and medial frontal cortex, consistent with models implicating age-associated changes in decision making and social cognition in financial exploitation [54]. Brain activity in the insula was furthermore specifically reduced in response to cues of untrustworthiness in older compared to younger adults [55], likely reflective of older adults’ reduced sensitivity to deceptive cues as the neurocognitive mechanism contributing to their poorer deception detection ability [53].

Of note, brain structural and functional influences on deception detection are exacerbated in pathological aging [28,29]. For example, a six-year longitudinal study among older adults who were free from dementia at baseline found that low scam awareness was associated with greater likelihood of developing Mild Cognitive Impairment, a risk factor of dementia, and higher burden of Alzheimer’s Disease pathology in the brain over time, with these associations persisted even after adjustment for global cognitive function [28]. Further, in amnestic mild cognitive impairment, lower cortical volumes in the angular gyrus [56] and lower medial and dorsal frontal cortex cortical volumes in early Alzheimer’s Disease [57] were related to reduced financial capacity.

## DECEPTIVE CONTEXTS

However, not only individual factors in psychological risk profiles but also variations in characteristics of deceptive messages contribute to deception detection and moderate the effectiveness of exploitation attempts (Figure 1; see also [14]). For example, deceptive messages can vary by the *weapons of influence* they use (i.e., prominent principles of influence/persuasion [11]) and/or by the life domains they refer to. In fact, a recent empirical analysis of modern spam found that the weapon of influence reciprocation (i.e., people’s tendency to repay, in kind, what another person has provided them) as well as the life domains of finances and leisure were overall most prevalent in email spam [58]. The study further revealed that types of spam received in email inboxes differed by age groups, with older adults receiving health and independence-related spam emails more frequently and younger adults receiving leisure and occupation-related emails more frequently, indicating targeted campaigns. Furthermore, a study using an ecologically-valid behavioral measure of email phishing susceptibility found that reciprocation was the most effective psychological weapon of influence among older adults, while younger adults were most likely to fall for phishing emails that employed scarcity (i.e., people’s tendency to perceive the value of an object, experience, or opportunity as greatly increased when its availability is limited/“soon-to-expire”) [18]. These findings combined emphasize the need for age-tailored warnings (instead of a one-size-fits-all approach) to enhance deception attempts as they vary across contexts.

Also, the *personal relevance* of deceptive cues has been shown to impact deception detection. Generally, self-relevant information (as opposed to information related to other people) is processed very efficiently (see [59] for a review) by activating unique brain regions (e.g., medial prefrontal cortex [60]). Along these lines, the perception of a computer virus warning topic as high (vs low) in personal relevance resulted in greater likelihood to believe a rumor about that computer virus [61]. Additionally, framing of a deceptive message as *gain vs loss* influences deception detection ability. For example, the intention of responding to a marketing solicitation was positively associated with perception of gains from an offer (e.g., potential of receiving a prize in sweepstakes scams [62]). In addition, highlighting the role of familiarity on deception detection accuracy, fake news detection was less accurate in both younger and older adults for less familiar news topics (e.g., COVID-19 related news vs. regular everyday news stories [24]). Further, reliability (i.e., a high number of Facebook likes in [63]) and credibility (i.e., reputation of news outlets) in [64]) of news sources have been found to enhance accuracy in news veracity judgments.

The presence and the content of *visual cues* have furthermore been shown to moderate the ease with which deception is detected. The psychological literature has especially looked into effects of facial trustworthiness in this regard [65,66]. In fact, facial cues are routinely used to evaluate trustworthiness, and even brief exposures to faces (i.e., 100 ms [67]) are sufficient to form trait inferences, often with behavioral consequences (e.g., voting [68]; deciding the guilt of a defendant [69]; criminal sentencing [70]). Sociobiological features of faces (sex, age, emotion, dominance, attractiveness [71,72]), often in tandem, moderate facial trustworthiness perceptions [73,74], with some variations between younger and older adults. For example, both age groups perceived older faces depicting negative emotions (e.g., disgust) as less trustworthy than younger and happy faces [75]. Older compared to younger adults, however, rated neutral untrustworthy-looking faces as more trustworthy and showed dampened amygdala response to them [76] (see also [77]). Using a novel dynamic trust-learning paradigm modeled after the Iowa Gambling Task [78,79], older relative to younger adults were less able to override an initial face bias (i.e., to select card decks represented by faces objectively rated as high in trustworthiness when those decks consistently resulted in negative financial outcomes [80]), supporting the notion that first impressions of facial trustworthiness bias behavior in aging by increasing older adults’ difficulty in detecting the “wolf in sheep’s clothing”, heightening vulnerability to fraud and exploitation.

Importantly, however, but not well addressed yet in research, good deception detection ability neither depends solely on individual characteristics of human targets nor solely on specific features of deceptive cues, but rather is a result of the interplay between the two (Figure 1). Supporting this notion, shared group membership (such as belonging to the same age group), for example, can enhance deception detection. In particular, older adults showed a greater bias toward thinking that older speakers were truth tellers in a lie detection task [81], whereas college-aged participants were better at detecting lies from younger adults [82]. Similarly, older (relative to younger) adults trust older trustees more than younger in economic trust games [83]. Also, older adults show reduced loss aversion, reflected in less insula and caudate activity in anticipation of losses (but not gains) [84], possibly reflecting reduced negative arousal in response to negative information in line with the “positivity bias” (i.e., a shift towards preferential processing of positive relative to negative information with age [85]).

## FUTURE RESEARCH DIRECTIONS

Moving forward, a promising future research direction is the study of the dynamic interplay of cognitive, socioemotional, and neurobiological mechanisms in humans when interacting with various deceptive cues. In this context, one of the most important tasks is the identification of reliable fraud susceptibility profiles, which will not only enhance surveillance of particular at-risk individuals but also inform the design of effective, tailored protection solutions. This work will require implementation of newly designed clinical tools (e.g., interviews, questionnaires, behavior-based tasks) for early detection of at-risk individuals and will benefit from rigorous statistical and novel machine-learning methodology (see [13,14] for discussions).

Future research will also benefit from extending breadth and depth of training interventions to enhancing deception detection ability and reducing exploitation risk, by ensuring longer-term training efficacy and success [86], which in older populations have been largely ignored. In particular, process-based approaches are particularly promising, such as by training higher-order cognitive operations (e.g., decision making under ambiguity) governed by executive control processes [87], given their proven efficiency and better transfer (e.g., to attention or memory processes) than strategy-based training focused on the detection of specific deceptive cues only (e.g., certain verbal or non-verbal content during communications [88,89]). Also, spaced and multi-session training may be particularly beneficial with aging populations based on successful approaches from the cognitive aging training literature [90].

Additionally, both cross-sectional and longitudinal evidence suggests that age-related deterioration in financial and health literacy, defined as the ability to acquire, manipulate, and utilize financial and health knowledge [91–94] contribute to vulnerability to scams with age [95–99]. For example, steeper age-related decline in financial and health literacy over time was associated with higher self-reported scam susceptibility among older adults [97]. In light of these findings, future efforts on designing interventions could benefit from incorporating training components that target financial and health literacy promotion to enhance complex decision making and reduce susceptibility to fraud among older adults.

Training efforts to improve socioemotional functioning in older adults have shown promise such as via supporting community-based engagement and physical activity to reducing loneliness [100,101], or interactive educational programs to improve emotional intelligence [102]. Finally, training is needed that targets neurobiological processes. Promising first evidence in this direction comes through training of interoceptive awareness by informing participants about the bodily signals they experience while watching emotional videos and their similarity to physiological sensations experienced during observing liars vs truth-tellers [43]. Such training resulted in enhanced lie detection compared to a control condition in which no information about the relation between physiological sensations of watching emotional videos and observing liars/truth-tellers was provided. These results encourage the development of novel bio- and neurofeedback training (e.g., heart-rate variability, real-time fMRI [103,104]), with demonstrated efficacy in aging, toward enhancing deception detection ability and reducing vulnerability to exploitation.

In addition, addressing racial and ethnic disparities in the prevalence of elder fraud is essential. Evidence suggests that older Black adults are frequent victims of financial fraud and exploitation [105]. Similarly, a 2016 Federal Trade Commission (FTC) report [106] indicated that Black and Hispanic consumers are disproportionately more likely to be victimized compared to non-Hispanic Whites. In 2011 alone, 17% of Blacks and 13% of Hispanics were victims, compared to 9% Whites; and the rate of complaints does not mirror the frequency of victimization due to underreporting especially among individuals from disadvantaged backgrounds [107]. Scam and fraud risks, especially online, have been further elevated during the COVID-19 pandemic, as public health guidelines on social distancing increased reliance on digital technology to stay connected. This may have particularly impacted minority elders with cognitive impairments, many of whom face isolation, limited resources, and lack of English fluency that further increase their vulnerability to misinformation and scams. These data underscore a strong need for reaching out to the underserved and developing culturally sensitive assessments and interventions that will resonate among diverse elders.

Leveraging fast-developing advances in artificial intelligence, effective defense solutions will be able to combine automated detection (e.g., spam filters, blacklists, face biometrics) and human decision making to overcome shortcomings in both the machine (e.g., attacks on spam filters [108]; false-positive categorization errors [109]; concept drift [110]) and the human mind (e.g., habituation [111]; overconfidence [112]; forgetfulness [113]; confirmation bias [114]). For example, deepfakes are images or videos created with artificial intelligence technology to fake someone’s audio-visual representation [115]. They have become a growing concern of deception on social and news media platforms [116]. While there have been some efforts to develop artificial intelligence-supported monitoring solutions, deepfakes are still capable of deceiving humans by bypassing artificial intelligence-based detection algorithms [117]. Only the combination of human deception and sophisticated artificial intelligence-guided detection systems can address these new deception detection challenges. Combined prevention and protection solutions could include interactive and educational gamified trainings such as proactive ‘inoculation’ approaches in which older adults are exposed to small doses of misinformation [118] or phishing material [119] to learn identification of suspicious information.

Finally, age-tailored defense solutions could be developed to offer on-the-spot warning about potentially deceptive messages (e.g., in SMS, email, social media platforms) using natural-language and image-based machine-learning algorithms. These applications will benefit from user-friendly, age-tailored designs and could be specifically based on individual risk profiles (e.g., more frequent and explicit warnings for older adults at higher risk). Such personalized automated cueing has tremendous potential to assist in real-time decision making, reducing the burden of detecting deception for the individual, and serving as an ad-hoc prevention solution for those particularly vulnerable to financial fraud in aging.

## CONCLUSIONS

In sum, financial exploitation is pervasive among individuals of all ages and across many contexts including face-to-face and online, constituting a public health crisis, with older adults representing a particularly vulnerable population due to age-related changes in cognition, socioemotional functioning, and neurobiology. The effects of financial exploitation can be devastating among older adults, contributing to deteriorating financial, social, and physical well-being. Research has made advances in identifying who is especially at risk in aging, knowledge that is crucial for individual tailoring and optimization of intervention to reduce deception and fraud among older adults. Most comprehensive has been research that considers the interplay between human characteristics with characteristics of fraudulent tactics and messaging itself. Leveraging such multifaceted approaches for risk profiling concurrently with state-of-the-art artificial intelligence detection technologies, age- and human-tailored protective software, and educational programs is promising in reducing vulnerability to fraud and deception among older adults.

## Data Availability

No new data was generated for the purpose of this mini review.
